# Structure and tailspike glycosidase machinery of ORF212 from *E. coli* O157:H7 phage CBA120 (TSP3)

**DOI:** 10.1038/s41598-019-43748-9

**Published:** 2019-05-14

**Authors:** Julia Greenfield, Xiaoran Shang, Heng Luo, Yan Zhou, Ryan D. Heselpoth, Daniel C. Nelson, Osnat Herzberg

**Affiliations:** 1 Institute for Bioscience and Biotechnology Research, University of Maryland, Rockville, Maryland USA; 2 Department of Chemistry and Biochemistry, University of Maryland, College Park, Maryland USA; 3 Department of Veterinary Medicine, University of Maryland, College Park, Maryland USA

**Keywords:** X-ray crystallography, Proteolysis, Biochemistry

## Abstract

Bacteriophage tailspike proteins mediate virion absorption through reversible primary receptor binding, followed by lipopolysaccharide or exopolysaccharide degradation. The *Escherichia coli* O157:H7 bacteriophage CBA120 genome encodes four distinct tailspike proteins, annotated as ORFs 210 through 213. Previously, we reported the crystal structure of ORF210 (TSP1). Here we describe the crystal structure of ORF212 (TSP3) determined at 1.85 Å resolution. As observed with other tailspike proteins, TSP3 assembles into a trimer. Each subunit of TSP3 has an N-terminal head domain that is structurally similar to that of TSP1, consistent with their high amino acid sequence identity. In contrast, despite sharing a β-helix fold, the overall structure of the C-terminal catalytic domain of TSP3 is quite different when compared to TSP1. The TSP3 structure suggests that the glycosidase active site resides in a cleft at the interface between two adjacent subunits where three acidic residues, Glu362 and Asp383 on one subunit, and Asp426 on a second subunit, are located in close proximity. Comparing the glycosidase activity of wild-type TSP3 to various point mutants revealed that catalysis requires the carboxyl groups of Glu362 and Asp426, and not of Asp383, confirming the enzyme employs two carboxyl groups to degrade lippopolysaccharide using an acid/base mechanism.

## Introduction

*Caudovirales*, the order of double-stranded tailed DNA bacteriophages, represents a diverse group of viruses with an estimated ten million species^1^. The *Caudovirales* is further divided into families based on tail morphology, one of which is the *Myoviridae* family characterized by a long contractile tail^2^. The tail machinery is responsible for host recognition during adsorption and subsequent injection of DNA into the host cell. Within the *Myoviridae*, the *Viunalikevirus* genus was initially established because all members shared a unique feature of possessing and displaying multiple, distinct tailspike proteins (TSPs)^3^. Sequencing of additional *Viunalikevirus* members and a more thorough analysis of their genomes resulted in renaming the *Viunalikevirus* as *Ackermannviridae* and promoting the genus to a new family under the *Caudovirales*^4^.

TSPs serve as primary receptor binding proteins that allow for the initial reversible contact between the phage and the bacterial cell. After binding to the bacterial lipopolysaccharide (LPS), TSPs exhibit glycosidase or esterase activity directed toward the receptor/substrate that allow the tail fibers or other receptor binding proteins, which lack enzymatic activity, to irreversibly bind secondary receptors to facilitate host invasion. TSPs form highly stable homotrimers, with each subunit divided broadly into two main domains. The N-terminal domain, also termed the head-binding domain, attaches the tailspike to the baseplate of the virus particle. An α-helical domain, termed the “neck”, connects the head-binding domain to the C-terminal receptor-binding domain. The receptor-binding domain is responsible for both receptor recognition and degradation, therefore it is also termed the catalytic domain.

Electron microscopy uncovered an unusual *Ackermannviridae* tail structure that differs from the long, spindly tail fibers of the T4-like myovirus. Instead, *Ackermannviridae* family members have an elaborate organization of branched projections coupled with bulbous prongs protruding from the base plate. These branched projections have been attributed to the TSPs^3^. Because each TSP may recognize a different receptor/substrate, it has been suggested that perhaps the *Ackermannviridae* members can infect a broader range of bacteria^5^. Indeed, infection of multiple bacteria species has now been described for the *Ackermannviridae* Sfp10^5^.

There is evidence that multiple unique TSPs from the same phage can form protein-protein interactions to yield higher-ordered structures. For example, phage vB_EcoP_G7C of *E. coli* 4 s, encodes two tailspikes, gp66 and gp63.1^6^. Interpretation of the electron microscopy images, sequence analysis, and truncation mutants led Prokhorov and colleagues to propose that gp66 attaches to the virus particle via a N-terminal 134-residue domain, whereas the gp63.1 head-binding domain binds the ensuing 150 amino acid of gp66 rather than directly to the baseplate. The 294-residue N-terminal region of gp66 is followed by two domains comprising canonical tailspike head- and receptor-binding domains^7^.

*Escherichia coli* phage vB_EcoM_CBA120 (CBA 120), a member of the *Ackermannviridae* family and the architype of the new *Cba120virus* genus, has four tailspikes encoded by ORFs 210 through 213, denoted herein as TSP1-4^3^. CBA120 was isolated from a cattle feedlot and infects enterohemorrhagic *E. coli* O157:H7^8,9^. The phage was shown to infect 13 of 17 *E. coli* strains bearing the O157:H7 serotype, but only one out of 77 non-O157:H7 strains tested from the *E. coli* Reference Collection^10^. The specificity of CBA120 towards *E. coli* O157:H7 is of particular interest because it may be developed as a potential new bio-indicator or biocontrol agent.

The branched arrangement emanating from the phage CBA120 baseplate^3^ may reflect an hierarchical assembly of its four tailspikes. Of note, sequence analysis of the CBA120 tailspikes reveals that TSP1 and TSP3 contain only head-binding (hereinafter the “head-binding” is referred to as the “head” domain, since it has become apparent that not every head-binding domain binds to the phage particle) and catalytic domains, whereas TSP2 and TSP4 contain ~170 and ~340 amino acid N-terminal regions, respectively, preceding their head and catalytic domains. Indeed, the crystal structure of TSP1 confirmed an overall trimeric architecture that includes each subunit possessing head and catalytic domains^11^. The high resolution crystal structure of TSP3 described herein also confirms the presence of only head and catalytic domains with a similar overall fold to that of TSP1. TSP3 structure analysis and subsequent activity assays allowed for the identification of the glycosidase active site, as well as the essential residues required for catalysis.

## Results and Discussion

### Protein oligomerization state, stability, and protease sensitivity

Analytical size-exclusion chromatography of purified TSP3 revealed a single homogeneous peak at ~270 kDa, suggesting that TSP3 forms oligomers in solution (the calculated monomer molecular weight is 68,662 Da for the 6X-His tagged protein), although the accuracy of the method is insufficient to determine the exact oligomeric state. The crystal structure reported here clearly shows that similar to all other tailspike proteins with known structures^12^, TSP3 forms a trimer.

**Figure 1:**
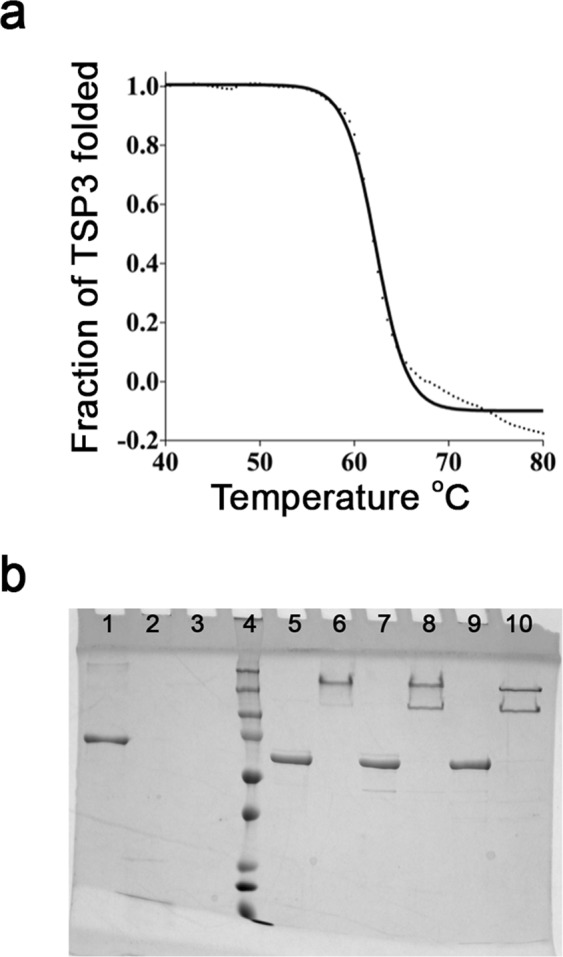
Biochemical and biophysical properties of TSP3. (**a**) Thermal unfolding followed by CD spectroscopy. The dotted curve corresponds to the experimental data and the solid line depicts the fit to sigmoidal function. The experimental data diverge from the sigmoidal curve at high temperature, suggesting aggregation. (**b**) LDS and protease susceptibility of TSP3. Lanes: BSA only (1); BSA + trypsin (2); BSA + chymotrypsin (3); protein molecular weight ladder (4); TSP3 + LDS, boiled (5); TSP3 + LDS, not boiled (6); TSP3 + trypsin, boiled (7); TSP3 + trypsin, not boiled (8); TSP3 + chymotrypsin, boiled (9) TSP3 + chymotrypsin, not boiled (10). Molecular weight markers from the lowest at the bottom to the highest at the top are as follows: 15 kDa, 20 kDa, 25 kDa, 37 kDa, 50 kDa, 75 kDa, 100 kDa, 150 kDa, 250 kDa.

Results from far-ultraviolet circular dichroism (CD) spectroscopy show TSP3 adopts an overall β-fold. When heated from 20 °C to 95 °C, the protein unfolded cooperatively with a T_m_ of 61.8 °C (Fig. 1a). Interestingly, the TSP3 T_m_ value is significantly lower than those reported for other tailspikes. High thermal stability of tailspikes, thought to be required to upstand harsh environmental conditions, has been reported in the literature for TSP1 of CBA120 (T_m_ = 80.7 °C), the TSP of P22 (T_m_ = 88.4 °C) and the TSP of HK620 (T_m_ = 80 °C)^11,13,14^.

Tailspikes are characteristically tolerant of anionic detergents and protease insensitive. To determine if this is true for TSP3, the protein was first treated with lithium dodecyl sulfate at room temperature or 100 °C for 10 min. Results from SDS-PAGE showed that TSP3 maintained its trimeric association when exposed to the detergent at room temperature (Fig. 1b, lane 6). Dissociation of TSP3 trimers into individual monomers required an additional boiling step after LDS treatment (Fig. 1b, lane 5). Next, protease susceptibility of TSP3 was determined. BSA, which is protease sensitive, was used as a control. Following 24 h incubation at 37 °C, the BSA control was completely digested by both tyrpsin (Fig. 1b, lane 2) and chymotrypsin (Fig. 1b, lane 3). Treating TSP3 with trypsin (Fig. 1b, lane 8) or chymotrypsin (Fig. 1b, lane 10) yielded two protein products, one corresponding to the full-length trimer and the second corresponding to an approximately 50 kDa lower molecular weight species. N-terminal sequencing of the protein extracted from both full-length and lower molecular weight bands revealed intact N-termini. Interestingly, after boiling the trypsin- (Fig. 1b, lane 7) and chymotrypsin-treated (Fig. 1b, lane 9) samples, a single band correlating to the full-length TSP3 monomer was observed. These observations can only be explained by a single cleavage site per trimer in a loop proceeding the head domain and neck, followed by dissociation and degradation of the cleaved catalytic domain, which has a molecular weight of ~50 kDa. Indeed, the structure determination shows that the linker contains solvent-exposed lysine and tyrosine residues (Lys176 and Tyr190, respectively). Interestingly, TSP1, which is resistant to 24 h proteolysis by trypsin and chymotrypsin has an analogous but shorter linker loop that does not contain positively charged or aromatic residues, the preferred residues for cleavage by trypsin and chymotrypsin, respectively^11^.

### Overall crystal structure

**Figure 2:**
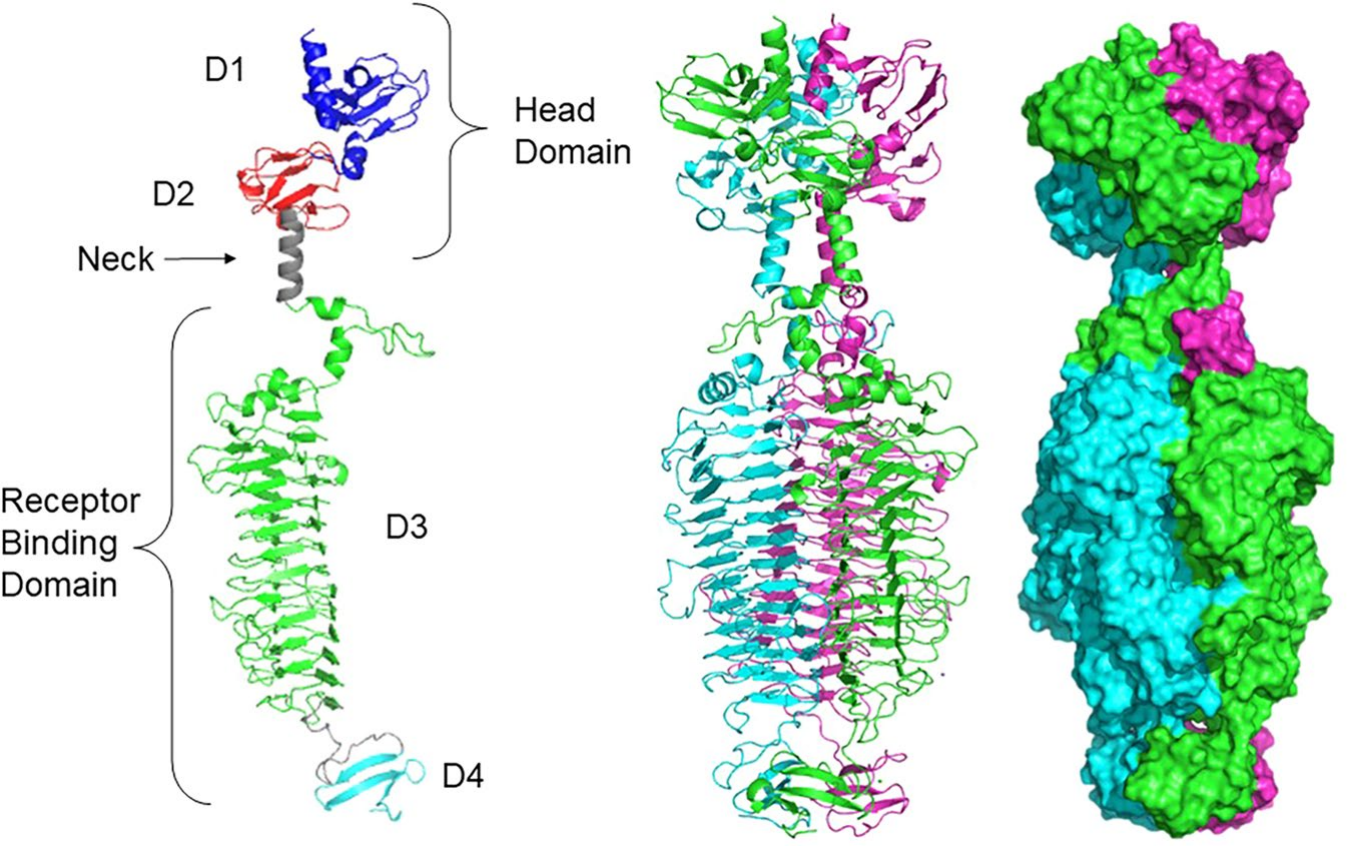
Overall structure of TSP3. A cartoon representation of the monomer (left), the trimer (middle), and the trimer surface representation of TSP3 (right). Each molecule of the trimer assembly is colored differently.

**Figure 3:**
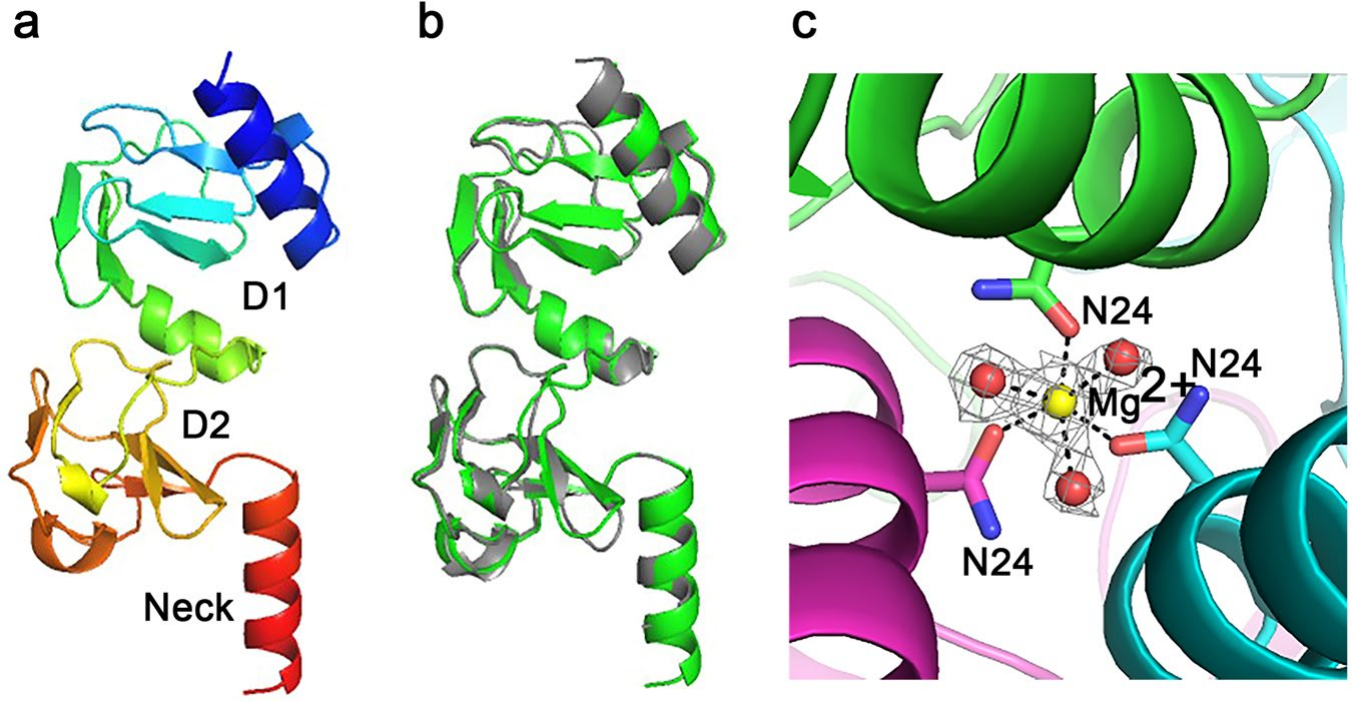
TSP3 head domain structure. (**a**) Cartoon representation of the two N-terminal subdomains and the neck with rainbow coloring from the N-terminus (blue) to the C-terminus (red). (**b**) Cartoon image of superimposed head and neck regions of TSP1 (gray) and TSP3 (green). (**c**) Mg^2+^ binds along the trimer 3-fold axis close to the C-terminus of the N-terminal 3-helix bundle with octahedral coordination to an Asn24 side chain from each subunit and three water molecules. The Mg^2+^-ligand distances vary between 2.1 and 2.2 Å. The electron density associated with the cation and coordinating water molecules is shown (2Fo-Fc) coefficients and 1σ level. Carbon atoms and secondary structure unit of each subunits are shown in different colors. Oxygens and nitrogen atoms are colored red and blue, respectively.

The TSP3 structure was determined by SAD using the peak wavelength at the Se absorption edge. The structure was refined at 1.85 Å resolution (Table 1). TSP3 consists of 627 amino acids followed by six C-terminal histidine residues engineered to facilitate affinity purification. The crystal asymmetric unit contains a biological homotrimer. The N-terminal 13–14 amino acids of each subunit have no associated electron density; thus, these were not modeled. The TSP3 trimer is an elongated structure, approximately 170 × 60 Å, which is broadly divided into the two canonical tailspike domains: (i) the N-terminal head domain (~25% of the polypeptide) and (ii) the C-terminal catalytic domain (~75% of the polypeptide) (Fig. 2). The N- and C-terminal domains of each subunit can be further divided into two subdomains analogous to those assigned to TSP1 structure; the D1 and D2 subdomains of the head domain, and the D3 and D4 subdomains of the catalytic domain^11^. D1 and D2 exhibit the same folds and spatial dispositions as those of the respective CBA120 TSP1 head subdomains (residues 13–96 and 97–154, respectively) (Figs 2 and 3). An ensuing α-helix (residues 155–168) links the head domain to the catalytic domain, with the α-helices of the three subunits forming the “neck” of the trimer. Subdomain D3, where the tailspike catalytic site resides, adopts a right-handed β-helix (residues 169–574) (Fig. 2). Following a 23 amino acid linker, a small D4 subdomain folds into a 3-stranded antiparallel β-sheet (residues 598–627) and the three D4 subunits are arranged in a β-prism II architecture (β-sheets perpendicular to the 3-fold axis), as first observed in the structure of a mannose-specific snowdrop lectin^15^. Hence, the overall folds of the D1, D2, and D3 subdomains are the same as those of the respective TSP1 subdomains, whereas the TSP3 D4 fold is different than the D4 β-helix fold of TSP1. Interestingly, the β-prism II architecture is also adopted by the D4 subdomains of the P22 and Det6 tailspike structures, although the β-sheets of these structures are flatter than those of the TSP3 D4^16,17^.

**Table 1:** Statistics of data collection, phasing, and refinement of CBA120 TSP3.

Data collection	Wild-type	Se-Met (absorption edge peak)
Wavelength (Å)	0.97932	0.97939
Resolution (Å)^a^	54.2-1.85 (1.92-1.85)	29.6-1.91 (1.94-1.91)
Space group	P 2_1_	P 2_1_
Unit cell dimension (Å, °)	a = 99.4, b = 66.8, c = 161.6,	a = 67.7, b = 117.6, c = 121.0,
	β = 103.6	β = 100.8
No. of molecules in the asymmetric unit	3	3
No. of unique reflections	175806 (17301)	140860 (6814)
Multiplicity^a^	2.0 (1.9)	2.6 (2.6)
Completeness (%)^a^	99.7 (99.1)	98.1 (95.8)
Mean I/σ(I)^a,b^	12.5 (4.8)	12.2 (1.6)
*R*_*merge*_^a^	0.048 (0.217)	0.058 (0.687)
**SAD-Phasing**		
Resolution (Å)		2.1
No. of Se atoms (found/correct)		29/19
CC (%)^c^		37.1
**Refinement**		
Resolution (Å)^a^	1.85 (1.871-1.850)	
Total no. of reflections^a^	175535 (5468)	
*R*_*work*_/*R*_*free*_^a,b^	0.152 (0.206)/0.184 (0.233)	
No. Protein residues	1851	
ligands	95	
solvent	1829	
RMSD from ideal geometry		
bonds length (Å)/bond angles (°)	0.012/1.4	
Ramachandran Plot: favored/allowed/outliers (%)	96.4/3.32.99/0.3	
Molprobity overall score & percentile^d^	1.4, 98%	

^a^The values in parentheses are for the highest resolution shell.

^b^*R*_*merge*_ = ∑_*hkl*_∑_*j*_|*I*_*j*_(*hkl*) − < *I*(*hkl*) > |/∑_*hkl*_ ∑_*j*_*I*_*j*_(*hkl*).

*R*_*work*_ = ∑_*hkl*_|*F*_*o*_ − *F*_*c*_|/∑_*hkl*_ *F*_*o*_, where *F*_*o*_ and *F*_*c*_ are the observed and calculated structure factors, respectively.

*R*_*free*_ is computed from 4.94% of randomly selected reflections (8672) and omitted from the refinement.

^c^Correlation coefficient between *E*_*c*_ and *E*_*o*_ in SHELXD^26^.

^d^Molprobity geometry score and percentile correspond to PDB structures within the refinement resolution range^34^.

### Head domain

The head domain of TSP3 shares 71% sequence identity with that of TSP1 and adopts the same overall fold with a root mean square deviation (rmsd) over 154 α-carbon atoms of 0.5 Å (Fig. 3b). The head domain also shares high sequence identity with several tailspike proteins from other phages including 97% identity with the *Salmonella* phage Det7 TSP^3,10^, whose head domain structure has not been determined, and 70% identity with TSP gp63.1 from the non-*Ackermannviridae* family member *E. coli* phage G7C, which adopts the same head domain structure but has an entirely different catalytic domain fold and enzymatic function (i.e., deacylation of the LPS)^7^. In addition to Det7, TSP3 head domain homologs of other members of the *Ackermannviridae* share sequence identities with that of the CBA120 TSP3 head domain as follows: *Dickeya* phage Limestone (73%), *Escherichia* phage PhaxI (80%), *Salmonella* phage PhiSH19 (97%), and *Salmonella* Phage ViI (94%). Accordingly, their head domains are expected to adopt the same structure as the CBA120 TSP3 head domain.

An N-terminal three α-helix bundle imbedded within the core of the D1 subdomain incorporates a metal ion close to the C-terminus of the bundle along the 3-fold axis of the trimer (Fig. 3c). The cation coordinates six ligands in an octahedral geometry (one Asn24 side chain from each subunit and three water molecules). The cation-ligand distances vary between 2.1 and 2.2 Å. The octahedral coordination and the cation-ligand distances are consistent with Mg^2+^. A Zn^2+^ binds to TSP1 at the same position, but with tetrahedral coordination to three histidine residues and a single water molecule^11^. In both TSP1 and TSP3, the cation coordination at the center of the trimer may contribute to the stability of the head domain.

### Catalytic domain

The helical neck, a characteristic of all tailspike structures, is followed by the catalytic domain (Fig. 2). The 61 N-terminal amino acid residues of subdomain D3 (residues 169–230) form two splayed loops that contain 1-turn, 2-turn, and 3-turn α-helices. This region wraps below the neck such that the loop flanked by the first two α-helices interdigitates between the neck and the D3 subdomain of a partner subunit to enhance subunit-subunit interactions, as observed with TSP1 and other tailspike structures (i.e. HK620 and Sf6 tailspikes)^13,18^. The 2-turn α-helices (Val193- Asp199) of the three TSP3 subunits form a α-helix bundle along the axis of the neck helical bundle with translational displacement and a tilt relative to the neck helices. The ensuing loop and 3-turn α-helix caps the D3 β-helix, traversing the center of the β-helix cross-section. A similar, although not identical, capping loop and α-helix has been observed in other tailspikes, including TSP1 and the tailspikes of P22, Det6, SF6, and HK620 phages^11,13,16–18^. Interestingly, the structure of pectate lyase, the founding member of the β-helix fold also contains a capping α-helix^19^.

The 3-sided β-helix (Fig. 2) comprises three β-sheets packed approximately in a triangular cross section beginning at residue 231. The β-helix contains 13 and 2/3 helical turns, as the last turn is incomplete. The linkers between β-strands vary in length. Those facing the trimer 3-fold axis are very short (1–3 residues), except for the two N-terminal β-helical turns, which are longer. In contrast, some of the linkers facing solvent are much longer. Notably, two long loops (residues 402–415 and residues 522–533) interact with one another across subunits. These loops block the cleft formed along the interface between two β-helices. The active site is located in the cleft close to these loops, as discussed in the next section.

**Figure 4:**
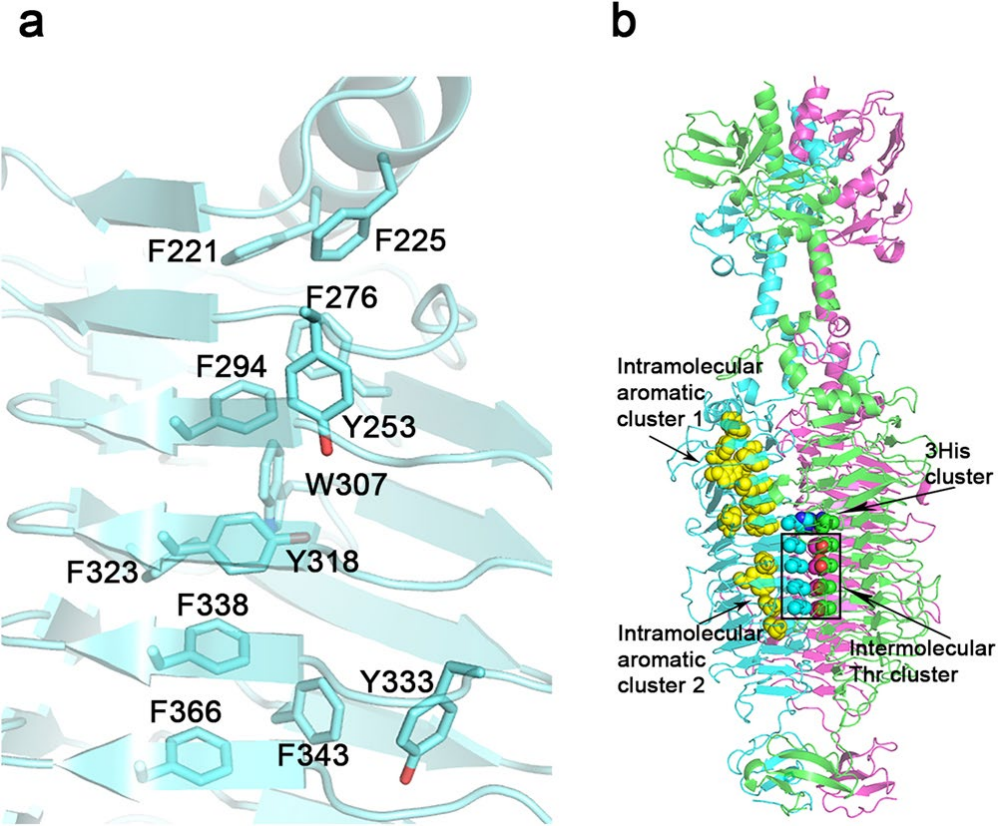
Intramolecular aromatic clustering in the catalytic domain of TSP3. (**a**) close-up of the larger cluster (cluster 1) comprising 12 aromatic amino acids (**b**) The two intramolecular clusters are highlighted as yellow spheres in the context of the entire trimer. An intermolecular threonine ladder and the preceding 3 His367 residues are also indicated.

Even though the D3 overall folds of TSP1 and TSP3 are similar, their structures are quite different in detail. Nevertheless, as with TSP1, the intramolecular core of each TSP3 β-helix is enriched with hydrophobic residues including stacking and edge-to-face aromatic side chains including Phe221 and Phe225 of the capping α-helix followed by Phe276, Tyr253, Phe294, Trp307, Tyr318, Tyr 333, Phe338, Phe348, Phe366, Phe425 (Fig. 4). A second cluster includes Trp433, Phe474, Phe492, Phe497, Tyr536, and Phe553 (Fig. 4b). Some residue placements are quite striking because of the tendency of the same side chain to stack one above the other along the β-helix rungs. For example, three cysteine residues (Cys341, Cys369, and Cys389) are positioned adjacently along three consecutive rungs of the internal β-sheet ladder, all adopting the same side chain staggered conformation so that the distances between thiol groups are too long for disulfide bonds. Notably, a positively charged amino acid in the hydrophobic core of the β-helix, Arg343, has no adjacent counter negative charge. Instead, the guanidinium group charge is countered by multiple electrostatic interactions with two backbone carbonyl oxygens (Ser374 and Pro375), a serine hydroxyl group (Ser371), and a water molecule. The guanidinium-ligand distances vary between 2.7 and 3.1 Å.

**Figure 5:**
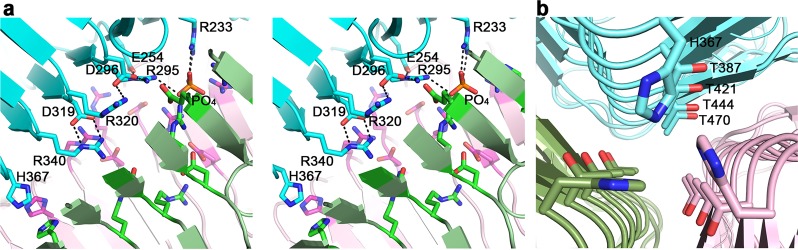
Intermolecular interactions along the trimer 3-fold axis of TSP3 catalytic domain. (**a**) Stereoscopic representation of intramolecular charge-charge interaction. For clarity, the residue labeling and interactions are shown for only one molecule, but the same interactions repeat in triplicates. (**b**) The stacking of threonine residues along the β-helix rungs proceeding His367. Again, residues labels are shown for only one molecule.

Intermolecular contacts along the trimer axis are mediated by both hydrophilic and hydrophobic interactions with many internal water molecules that solvate the hydrophilic residues. On the sixth β-helix rung, the three His367 side chains stack edge to face across the 3-fold axis (Figs 4b and 5). The three interacting water molecules each form a hydrogen bond to one of these histidine residues. An exquisite salt bridge network along the rungs N-terminal to the His367 cluster includes Arg233, Arg295, Arg320, Arg340, Glu254, Asp296, and Asp319 (Fig. 5a). In addition, electron density consistent with a tetrahedral ion was assigned as PO_4_^−3^ as the crystallization solution did not contain ammonium sulfate. The phosphate ion coordinates the three Arg295 and three Arg233 side chains at the N-terminus of the β-helix. Multiple water molecules surround the charge network. On a β-helix His367 cluster rung and the adjacent rung, Lys365 and Lys388 each interacts with a neighboring molecule via a backbone carbonyl oxygen and a threonine O^γ^. Another striking structural feature following the His367 cluster is the stacking of 4 threonine residues (Thr387, Thr421, Thr444, and Thr470) on sequential rungs of the internal β-sheet ladder, each on the C-terminus of a β-strand (Figs 4b and 5b). All five ensuing turns along the internal trimer interface, on the rungs containing His367 and the four threonine residues listed above, as well as the turns of the rungs preceding His367, have the same conformation.

The 39 amino acid D4 subdomain of TSP3 is one of the smallest when compared to other TSP structures. Furthermore, the fold of the D4 subdomain of TSP3 is very different than that of its TSP1 counterpart. A long D3-D4 linker traverses the β-helix so that D4 is rotated with respect to D3 by approximately 45° and each D4 β-sheet plane intersects two β-helices (Fig. 2, middle). The core of the D4 β-prism II is lined with three asparagine, tryptophan, and proline residues.

### Active site

Despite the shared β-helix fold of the tailspike glycosidase domains, the locations of the active sites vary. For example, the active sites of the tailspikes from phages P22, HK620, and Det7 are located intramolecularly in a shallow depression formed by large loops on the surface of the solvent-exposed β-sheet of each β-helix subunit. Conversely, the active site of the phage Sf6 tailspike is located in a cleft along the interface between two β-helix subunits^13,17,18,20^. Nevertheless, the glycosidase catalytic machinery of these tailspikes always employs the carboxyl groups of two neighboring Asp/Glu amino acid residues, consistent with an acid/base mechanism^21^. Koshland described two glycosidase acid/base mechanisms. The first involves two steps; cleavage of the glycosidic bond followed by a water molecule replacing the leaving aglycon. This mechanism results in retention of stereochemical configuration at the anomeric carbon. With the second mechanism, the hydrolytic water molecule binds between the substrate and the base, enabling cleavage of the glycosidic bond to proceed concomitantly with the water attack resulting in inversion of configuration of the anomeric carbon^21^.

**Figure 6:**
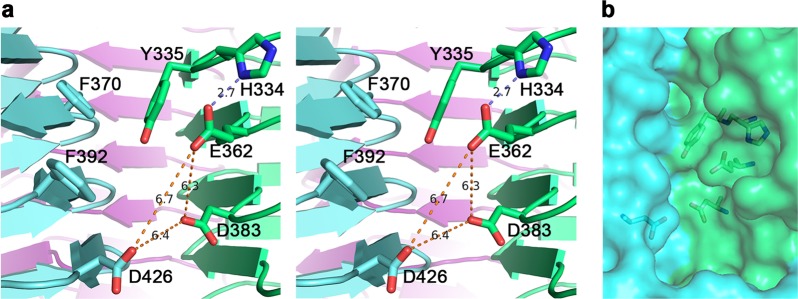
The active site of TSP3. (**a**) Stereoscopic representation of active site residues. The distances between the three carboxyl groups of Glu362, Asp383, and D426, the candidate catalytic resides that were probed by site-directed mutagenesis, are shown. The hydrogen bond between Glu362 and His334 is also highlighted. Aromatic groups may interact with the substrate pyranose rings. The trimer subunits are colored differently. (**b**) The partially transparent molecular surface at the active site depicted in the same orientation as in (**a**) and highlighting key active site residues. As can be seen, the interface between the green and cyan molecules forms a cleft. The trimer contains three such clefts at the three subunit interfaces.

Examination of the TSP3 crystal structure did not reveal intramolecular surface pockets flanked by pairs of carboxyl groups where the substrate could bind and be cleaved. However, Glu362 and Asp383 on one subunit and Asp426 on the adjacent subunit are in close proximity within the intermolecular cleft (Fig. 6). The question was which pair of the three Asp/Glu residues was indispensable for catalysis. The distances between each of the possible carboxylate pairs vary between 6.2 ± 0.1 Å for the three Glu362-Asp383 pairs of the trimer, 6.5 ± 0.2 Å for the Asp383-Asp426 pairs, and 6.8 ± 0.1 Å for the Glu362-Asp426 pairs. By comparison, the two carboxylate groups of hen egg lysozyme, a “retaining” enzyme, are 6.2 Å apart in the 0.92 Å resolution structure (PDB entry 3LZT), whereas those of bacteriophage T4 lysozyme determined at 1.45 Å resolution, which is an “inverting” enzyme, are 7.2 Å apart (PDB entry 4W51). Thus, in contrast to the Koshland model that stipulated carboxy-carboxy distances of ~5 Å and ~10 Å for the retaining and inverting mechanisms, respectively, the experimental distances observed in high resolution crystal structures may differ by as little as 1 Å between the two mechanisms. This is because the hydrolytic water molecule in the inverting mechanism needs not be placed between the anomeric carbon and the base as long as it is oriented appropriately for nucleophilic attack close to the sugar anomeric carbon and is close enough to the base to be activated by the negative charge.

Aromatic residues line the interface along the solvent-exposed edge of the cleft. Those closest to the postulated active center are Tyr335 and Phe392, each on a neighboring molecule (Fig. 5). Other, more remote aromatic residues that line the edge of the cleft include Phe370, Trp344, Tyr324 and Phe322. Aromatic residues are known to stack against sugar pyranose rings, thus the disposition of aromatic side chains along the intermolecular cleft may assist with orienting the LPS chain appropriately for catalysis.

### Identification of the TSP3 catalytic residues

**Figure 7:**
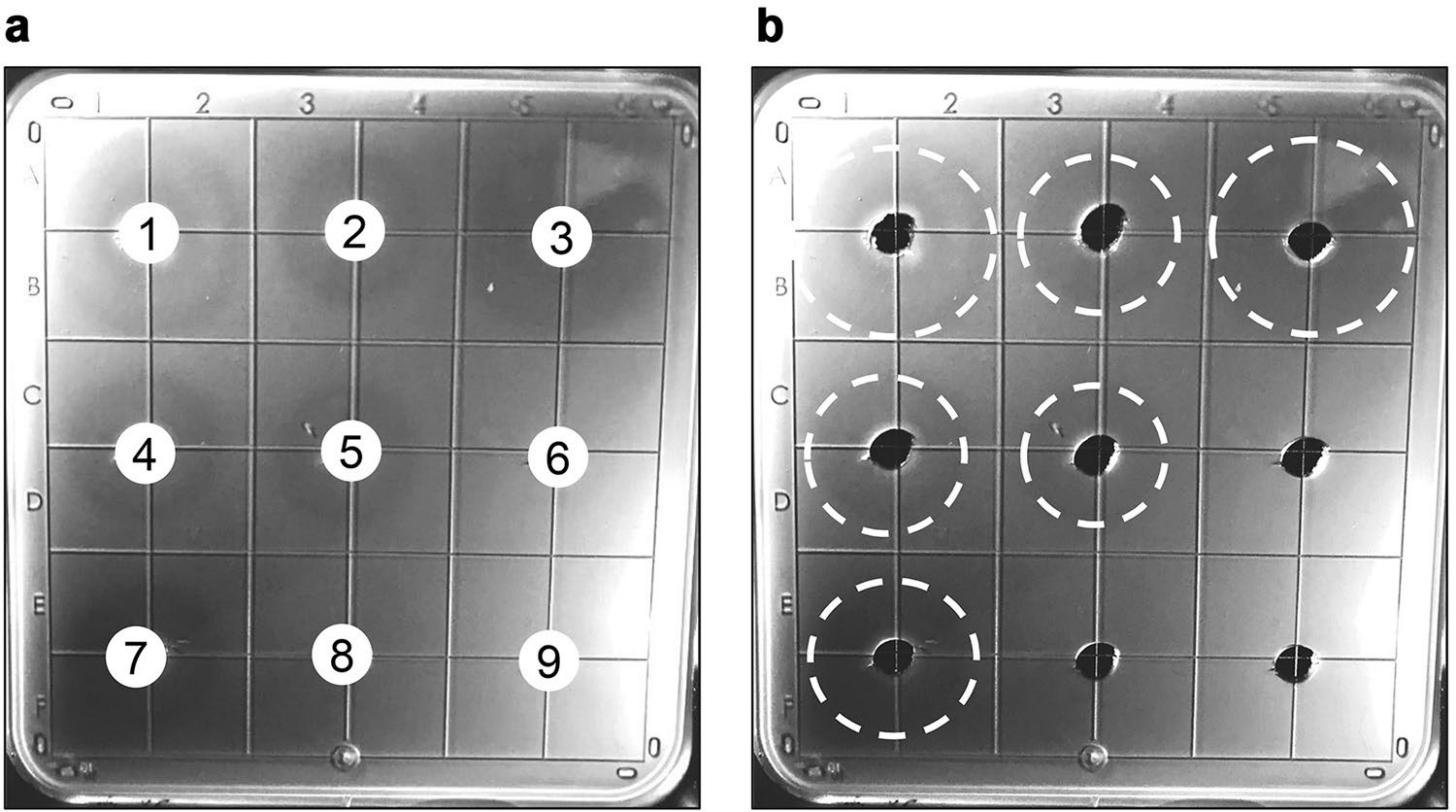
Active site mutant halo assays. *E. coli* strain ATCC 700728 was embedded in agarose. Wells (3 mm) were cut out of the agarose and loaded with 10 µL (6 mg/mL) wild-type TSP3 or active site mutants and incubated overnight to visualize tailspike activity. (**a**) (1) wild-type TSP3; (2) TSP3 E362Q; (3) TSP D383N; (4) TSP3 D426N; (5) TSP3 E362Q:D383N double mutant; (6) TSP3 E362Q:D426N double mutant; (7) TSP3 D383N:D426N double mutant; (8) TSP3 E362Q:D383N:D426N triple mutant; (9) PBS control. (**b**) Identical image to (**a**) with a dashed white line to indicate the halo edge to aid in visualization. Quantitation of Fig. 6 is shown in Table 2.

In their role as primary receptor binding proteins, tailspikes are known to bind to, and often cleave or modify, the bacterial LPS. Although tailspikes do not lyse bacterial cells, the thinning of the LPS layer can be viewed as a halo of less density or less opacity on an agar dish embedded with sensitive bacteria. Whereas this does not address the specific glycosidic cleavage site, it does allow for semi-quantitative demonstration and comparison of activity. With this in mind, single, double, and triple replacements of Glu362, Asp383, and Asp426 in TSP3 were generated by site-directed mutagenesis. Using the halo assay, equal weighted amounts of purified wild-type and mutant TSP3 proteins were analyzed (Fig. 7, Table 2) in order to determine which amino acid residues comprise the catalytic machinery. As expected, a halo was observed when spotting wild-type TSP3 (Fig. 7, well 1), while the buffer only negative control (Fig. 7, well 9) was devoid of activity. Similar to the negative control, the TSP3 E362Q:D383N:D426N triple mutant (Fig. 7, well 8) was incapable of generating a halo, implying glycosidase activity was abolished and thus confirming the location of the active site. Individual TSP3 point mutants were assayed next in order to identify which specific residues are required for catalysis. When compared to wild-type, the TSP3 D383N mutant (Fig. 7, well 3) displayed comparable activity, indicating D383 is dispensable for activity, whereas the E362Q (Fig. 7, well 2) and D426N (Fig. 7, well 4) mutants exhibited activity defects. Contrary to E362Q:D383N (Fig. 7, well 5) and D383N:D426N (Fig. 7, well 7), the activity of the TSP3 E362Q:D426N double mutant (Fig. 7, well 6) was abolished. This result suggests the two essential catalytic residues of TSP3 are E362 and D426, located on two adjacent subunits. The crystal structure shows that the distance between these two carboxylate groups is 6.8 Å, a distance that does not distinguish between “inverting” and “retaining” glycosidase mechanisms.

**Table 2:** TSP3 active site mutant halo assay results.

Well^a^	Mutation	Halo Formation^b^
1	Wild-type	**++**
2	E362Q	**+**
3	D383N	**++**
4	D426N	**+**
5	E362Q and D383N	**+**
6	E362Q and D426N	**−**
7	D383N and D426N	**+**
8	E362Q, D383N, and D426N	**−**
9	PBS Control	**−**

^a^Well numbering corresponds to Fig. 6.

^b^Symbols are as follows: −, No observable halo;+, 0–1.5 cm halo diameter; ++, >1.5 cm halo diameter.

**Figure 8:**
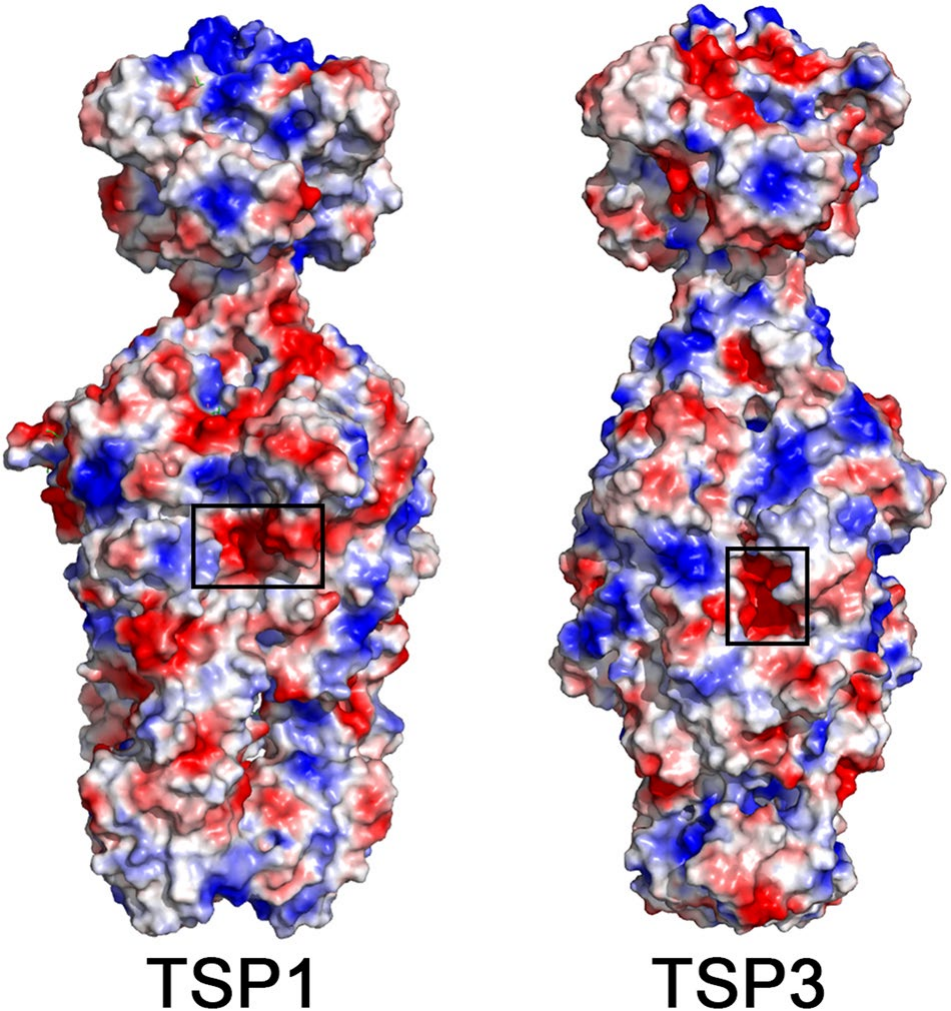
The location of the confirmed TSP3 and postulated TSP1 active sites. The molecular surface of the trimer is colored according to the vacuum electrostatic potential calculated with PyMol with red color depicting negatively charged regions and blue color depicting positively charged regions. The active sites are located in negatively charged pockets along grooves formed at the subunit interfaces.

The crystal structure of TSP1 suggested that similarly to TSP3, the active site is also located in a pocket formed between the trimer subunits (Fig. 8)^11^. The pocket contains acidic amino acids, as evident from the vacuum electrostatic potential (calculated with PyMol). We previously proposed that two adjacent glutamic acids that share a proton (Glu456-Glu483) could play a key role in a substrate-assited catalysis^11^. Another acidic residue on the neighboring subunit, ~10 Å away from the glutamic acid pair (Asp313), was not considered at the time, but perhaps could also be involved in catalysis. As of this writing, we have not identified the catalytic residues of TSP1 experimentally. Nevertheless, Fig. 8 shows that the postulated TSP1 active site pocket is displaced along the subunit interface with respect to the confirmed active site pocket of TSP3. In addition, the orientations and positions of the proposed TSP1 catalytic residues differ vastly from those of TSP3. Moreover, the arrangements of surrounding residues that are likely to support catalysis are also different. Taken together, the architecture of the active sites suggests that the catalytic machinery of TSP1 and TSP3 are likely to be quite different even though both may proceed via the acid/base mechanism.

### TSP3 specificity

By expanding the halo assay to examine other bacterial strains and species, it was found that TSP3 forms halos on both *E. coli* O157:H7 strains tested (ATCC 7000728 and ATCC 43894). In contrast, TSP3 could not form halos when applied to either a non-O157:H7 strain of *E. coli* (ATCC 35218) or other Gram-negative bacterial species (*K. pneumoniae*, *P. aeruginosa*, *A. baumannii*). Although the data initially suggested TSP3 displays specificity for the O157:H7 antigen, this tailspike was also capable of inducing halos when using the *E. coli* mutants TEA023, an O157:H7 knockout mutant of the *galU* gene, and TEA026, an O157:H7 knockout mutant of the *galETKM* operon^22^. Because the O157 antigen requires modification of galactose by the *galE*, *galT*, *galK*, and *galU* gene products, these mutants have been shown to lack the O157 O-antigen. Taken together, TSP3 glycosidase activity does not appear to require the presence of the O157 antigen. Whether TSP3 acts on outer core or inner core moieties of LPS remains to be determined. Likewise, it remains elusive which of the remaining three TSPs provides O157:H7 specificity for CBA120.

## Materials and Methods

### Bacterial strains

Most bacterial strains (Table S1) were purchased from the American Type Culture Collection (ATCC) or other commercial vendors. These include toxigenic (43894) and non-toxigenic (700728) strains of *E. coli* O157:H7, non-O157:H7 *E. coli* strains (35218 and BL21), and representative Gram-negative organisms (*Klebsiella pneumoniae* strain 700603, *Pseudomonas aeruginosa* strain 27853, and *Acinetobacter baumannii* strain BAA-1605). *E. coli* strains TEA023 and TEA026 are *∆glaU* and *∆ETKM* knockouts, respectively, of the O157 antigen in the EDL933 background and were obtained from Matthew Waldor’s laboratory^22^. All *E. coli* and *K. pneumoniae* strains were grown in Luria broth while *P. aeruginosa* and *A. baumannii* were grown in tryptic soy broth. Glycerol stocks of all strains were stored at −80 °C.

### TSP3 cloning, expression and purification

The nucleic acid sequence of TSP3 was codon optimized for expression in *E. coli*, modified to add a C-terminal 6X-His tag, and synthesized by GeneArt (Invitrogen). The gene was sub-cloned into a pBAD24 expression vector, and the resulting clone was used to transform Rosetta-gami™ 2 competent cells^23^. The transformed cells were used to inoculate 2x yeast extract tryptone (YT) medium supplemented with 200 μg/mL ampicillin at 37 °C for 4–8 hours until the OD_600_ reached 0.8. The temperature was lowered to 18 °C for induction with 0.25% (w/v) arabinose and the biomass was collected after overnight growth by centrifugation. The pellet was resuspended in PBS buffer, lysed by sonication, and centrifuged at 14,000 rpm for 45 min. The resulting supernatant was incubated with Ni-NTA agarose for 1 h at 4 °C. TSP3 was purified by Ni affinity gravity-flow chromatography, and dialyzed in Tris-HCl buffer (pH 7.5). Production and purification of TSP3 containing selenomethionine (SeMet) followed the same protocol except that the cells were grown in SeMet medium (Molecular Dimensions Limited) supplemented with 40 mg/L SeMet.

### Site-directed mutagenesis

Plasmids (Table S2) harboring the TSP3 mutants were constructed using the QuikChange II Site-Directed Mutagenesis Kit (Agilent Technologies). Primers (Table S3) were designed to include mutations in the middle of 30 nucleotide phosphorylated forward primer, with the phosphorylated reverse primers complementary to the next 30 nucleotides upstream. The resulting PCR products were digested by *DpnI* to remove the methylated templates, ligated and transformed into *E. coli* DH5ɑ. The mutations were confirmed by nucleotide sequencing (Macrogen, USA) before being transformed into *E. coli* BL21(DE3) for protein expression.

### Analytical size-exclusion chromatography

The multimeric state of recombinant TSP3 was determined by analytical size-exclusion chromatography. The protein was applied to a pre-equilibrated Superose 6 column (GE Healthcare) and run under isocratic conditions in PBS for 1.5 column volumes on an AKTA FPLC system (GE Healthcare). The molecular mass of TSP3 was estimated from a standard curve generated by linear regression of log (molecular mass) vs. retention volume using gel filtration standards (Bio-Rad).

### Thermal stability measurements

The thermal stability of TSP3 was investigated using a Chirascan CD Spectrometer (Applied Photophysics). TSP3 (0.1 mg/mL) in 20 mM sodium phosphate buffer (pH 7.0) was gradually heated at a rate of 1 °C/min from 20 °C to 95 °C. The mean residue ellipticity of the sample contained in a quartz cuvette of 1 mm path length was monitored every 0.5 °C at 218 nm with 5 s signal averaging per data point. The melting temperature (T_m_) was calculated using the Pro-Data software (Applied Photophysics) based on data that was fitted to a Boltzmann sigmoidal curve.

### Susceptibility to anionic detergent and proteolysis

The sensitivity of TSP3 to anionic detergent was assayed by incubating the protein at 0.25 mg/mL with lithium dodecyl sulfate for ten minutes at room temperature or 100 °C. The structural integrity of TSP3 was then qualitatively analyzed by SDS-PAGE. To analyze TSP3 susceptibility to proteolysis, 0.25 mg/mL TSP3 was incubated in 20 mM sodium phosphate (pH 7.0) supplmented with 1 mM CaCl_2_ at 37 °C for 24 h with and either trypsin or chymotrypsin (Sigma Aldrich) at a 1:25 (w/w) protease:TSP3 ratio. Bovine serum albumin (BSA, Sigma Aldrich) served as a control. Degradation of the protease-treated samples was analyzed by 7% SDS-PAGE.

### Crystallization and structure determination

Crystals of wild-type and SeMet-containing TSP3 were obtained by the vapor diffusion method in sitting drops at room temperature. The reservoir solutions contained 18% (w/v) polyethylene glycol 8000, 0.2 M NaCl, and 0.1 M CHES (pH 9.0–9.8). Rod-shaped crystals appeared within a few days. Crystals were cryoprotected by adding to the drops equal volumes of reservoir solution supplemented with 30% (v/v) glycerol, transferring the crystals to mounting pins and flash cooling in liquid nitrogen. X-ray diffraction data were collected at beamline 23-ID_B managed by the General Medical Sciences and National Cancer Institute collaborative access team (GM/CA-CAT) at Argonne National Laboratory. The beamline was equipped with a MARmosaic MX-300 detector (Marresearch GmbH). Single anomalous diffraction (SAD) data for a SeMet TSP3 crystal was acquired at the Se absorption edge peak (0.97939 Å). The diffraction data of both wild-type and SeMet TSP3 crystals extended to a resolution of 1.85 Å and 1.91 Å, respectively. The data sets were processed using IMOSFLM^24^. The phases were determined by SAD using the HKL3000 software, which incorporates the programs SHELXD and SHELXE for heavy atom search^25–27^. As the wild-type TSP3 crystal form differed from the crystal form of the SeMet protein, Molecular Replacement to place the TSP3 in the wild-type crystal cell was performed using Phaser^28^ as implemented in CCP4^29^. The quality of the electron density map enabled most of the polypeptide chains to be built automatically using AutoBuild^30^. Structure refinement was performed with the Phenix software suite^31,32^. Structure modification was carried out using the interactive graphics computer program COOT^33^. Structure figures were prepared using the program PyMol (Schrödinger, LLC).

### Halo assays

For routine assays, a non-toxigenic strain of *E. coli* O157:H7 (ATCC 700728) was used. Bacterial strains were grown overnight at 37 °C with aeration to an OD_600_ = 1.6. After overnight growth, the bacterial cells were harvested via centrifugation at 4,150 rpm for 10 min at 4 °C. The cell pellets were washed twice using sterile PBS buffer and resuspended in buffer at 1/50 of the original volume. Next, 500 µL of concentrated bacterial cells were mixed with 10 mL of sterile 0.7% (w/v) agarose solution and plated in a square disposable petri dish with grid lines (Fisher Scientific). Holes (wells) with diameters of ~3 mm were generated on the solidified agarose using sterile plastic dropper tips (Fisher Scientific). 10 microliters of either wild-type TSP or its active site mutants were added to each hole at a concentration of 6 mg/mL. PBS buffer served as the negative control. Petri dishes were incubated for 24 h at 37 °C. Halos were visualized by holding the petri dish to a light box and photographing with a 12-megapixel camera (iPhone® 6S Plus). Contrast and brightness adjustments were applied to the entire image using Photoshop CC (Release 19.1.16, Adobe, Inc.) Clearing zones correspond to glycosidase activity.

## Supplementary information

Supplementary material

## Data Availability

The coordinates and structure factors were deposited in the Protein Databank, entry code 6NW9.
