# Supplementary material for: Structure and tailspike glycosidase machinery of ORF212 from E. coli O157:H7 phage CBA120 (TSP3)
Source: Sci Rep. 2019 May 14;9:7349. doi: 10.1038/s41598-019-43748-9 (PMC6517402; doi:10.1038/s41598-019-43748-9)
Supplement: Supplementary file 1 — Supplementary material [file 41598_2019_43748_MOESM1_ESM.pdf]

Structure and tailspike glycosidase machinery of ORF212 from *E. coli* O157:H7 phage CBA120 (TSP3)

Julia Greenfield[1,2], Xiaoran Shang[1], Heng Luo[1], Yan Zhou[1], Ryan D. Heselpoth[1], Daniel C. Nelson[1,3,*], Osnat Herzberg[1,2,*]

[1]Institute for Bioscience and Biotechnology Research, University of Maryland, Rockville, Maryland
[2]Department of Chemistry and Biochemistry, University of Maryland, College Park, Maryland
[3]Department of Veterinary Medicine, University of Maryland, College Park, Maryland

*Corresponding authors: nelsond@umd.edu; osnat@umd.edu

Supplementary Material

Table S1: Bacterial strains used in this study

**Organism**	**Strain**	**Source**	**Notes**
Escherichia coli	Rosetta-gami	Novagen	
Escherichia coli	700728	ATCC	O157:H7<br>Non-toxigenic strain
Escherichia coli	43894	ATCC	O157:H7 Toxigenic strain
Escherichia coli	35218	ATCC	
Escherichia coli	TEA023	Ref. 11	O157:H7 knockout mutant<br>EDL933 ΔgalU::aad-7
Escherichia coli	TEA026	Ref. 11	O157:H7 knockout mutant<br>EDL933 ΔgalETKM::aad-7
Klebsiella pneumoniae	700603	ATCC	
Pseudomonas aeruginosa	27853	ATCC	
Acinetobacter baumannii	BAA-1605	ATCC	

Table S2: Plasmids used in this study

**Plasmid**	**Relevant properties**	**Source or Reference**
pBAD24	Cloning and expression containing the arabinose pBAD promoter	Qiagen
pBAD24::*TSP3*	WT TSP3	This work
pBAD24::$TSP3_{E362Q}$	Site-direct mutagenesis of the 362nd amino acid, glutamic acid to glutamine	This work
pBAD24::$TSP3_{D383N}$	Site-direct mutagenesis of the 383rd amino acid, aspartic acid to asparagine	This work
pBAD24::$TSP3_{D426N}$	Site-direct mutagenesis of the 426th amino acid, aspartic acid to asparagine	This work
pBAD24::$TSP3_{E362Q\ D383N}$	Site-direct mutagenesis of the 362nd amino acid, glutamic acid to glutamine, and 383rd amino, acid aspartic acid to asparagine	This work
pBAD24::$TSP3_{E362Q\ D426N}$	Site-direct mutagenesis of the 362nd amino acid, glutamic acid to glutamine, and 426th amino acid, aspartic acid to asparagine	This work
pBAD24::$TSP3_{D383Q\ \mathrm{D426N}}$	Site-direct mutagenesis of the 383rd amino acid, aspartic acid to asparagine, and 426th amino acid, aspartic acid to asparagine	This work
pBAD24::$TSP3_{E362Q\ D383N\ D426N}$	Site-direct mutagenesis of the 362nd amino acid, glutamic acid to glutamine, 383rd amino acid, aspartic acid to asparagine, and 426th amino acid, aspartic acid to asparagine	This work

Table S3: Primers used in this study.

Purpose	Template	Sequence (5' > 3')*
Site-directed mutagenesis E326Q	pBAD24::*TSP3*	[Phos]-ATTCAGTTTATTAGC**CAG**AGCCCGAAATTTCAC
Site-directed mutagenesis D383N	pBAD24::*TSP3*	[Phos]-GTTTGGGATACCGCA**AAC**CTGGCAATTACCAAA
Site-directed mutagenesis D426N	pBAD24::*TSP3*	[Phos]- ACCTGTAGCGTTTTT**AAC**GCCGTTCGTGGTCAT
Site-directed mutagenesis E326Q D383N	pBAD24::$TSP3_{E362Q}$	[Phos]- GTTTGGGATACCGCA**AAC**CTGGCAATTACCAAA
Site-directed mutagenesis E326Q D426N	pBAD24::$TSP3_{E362Q}$	[Phos]- ACCTGTAGCGTTTTT**AAC**GCCGTTCGTGGTCAT
Site-directed mutagenesis E326Q D383N D426N	pBAD24::$TSP3_{E362Q\ D383N}$	[Phos]- ACCTGTAGCGTTTTT**AAC**GCCGTTCGTGGTCAT

* Point mutations bold
